# Service Evaluation of a Specialist Inpatient Psychiatric Rehabilitation Unit: Activity, Outcomes, and Occupancy Trends at Maple House Rehabilitation Unit, 2018–2024

**DOI:** 10.1192/bjo.2026.11546

**Published:** 2026-06-30

**Authors:** Vishwanath Byregowda Ramakrishna

**Affiliations:** Krinvest Healthcare Group, Warrington, United Kingdom

## Abstract

**Aims:**

To evaluate patient activity, clinical outcomes, and occupancy trends in a specialist inpatient psychiatric rehabilitation service over six years, with the goal of informing service delivery and quality improvement. The service (Maple House, Warrington) is a 23 bed high dependency rehabilitation unit for men with complex mental health needs due to mental illness and/or acquired brain injury.

**Methods:**

A retrospective evaluation of routinely collected clinical data from April 2018 to April 2024 was undertaken. Key indicators included referrals, admissions, discharges, length of stay (completed and ongoing cases), patient demographics, diagnostic profiles, Mental Health Act status, discharge destinations, and clinical outcomes. Descriptive statistics were calculated, including median and mean length of stay, admission and discharge rates, and outcome distributions. Ongoing occupancy and patient length of stay were analyzed to identify trends in service utilization.

**Results:**

- Referrals and Admissions: 76 referrals were received, of which 38 were admitted (50%).

- Discharges: 23 patients were discharged during the period (42.4% of admissions). Completed cases had a median length of stay of 746 days (approximately 25 months), in line with the planned treatment and rehabilitation pathway of 18–24 months.

- Demographics and Diagnoses: Patients were men, mainly in the age group of 20-40 years, with complex psychiatric conditions, including schizophrenia (51%), Schizoaffective disorder(11%), intellectual disability/Autism(11%), bipolar affective disorder(7%), organic personality disorder due to acquired brain injury(7%), and comorbid substance misuse in 26% of cases.

- Clinical Outcomes: Most patients showed measurable improvements in functional abilities, risk reduction, and engagement, with 70% discharged to supported or independent living.

- Occupancy Trends: The unit frequently reached full capacity, with several ongoing cases exceeding three years of admission as of April 2024, highlighting challenges in managing long-stay patients.

**Conclusion:**

The service delivers structured psychiatric rehabilitation for patients with complex needs, achieving clinical, functional and risk reduction outcomes largely within the intended treatment timeframe. Findings highlight areas for ongoing monitoring, including long-stay cases and occupancy pressures. This study is relevant considering the ongoing ACER study (Killaspy et al., 2021–2026) investigating the clinical and cost-effectiveness of in patient rehabilitation across the UK, reinforcing the need for further systematic evaluations in this area.

